# Effects of Parasitism and Venom from the Endoparasitoid *Brachymeria lasus* on Immunity of the Host *Galleria mellonella*

**DOI:** 10.3390/insects16080863

**Published:** 2025-08-19

**Authors:** Lijia Peng, Bo Yuan, Jiqiang Song, Fang Wang, Qi Fang, Hongwei Yao, Gongyin Ye

**Affiliations:** State Key Laboratory of Rice Biology and Breeding, Ministry of Agriculture and Rural Affairs Key Laboratory of Molecular Biology of Crop Pathogens and Insect Pests, Zhejiang Key Laboratory of Biology and Ecological Regulation of Crop Pathogens and Insects, Institute of Insect Sciences, Zhejiang University, Hangzhou 310058, China; 22216260@zju.edu.cn (L.P.); yuanbo_chn@zju.edu.cn (B.Y.); jqsong@zju.edu.cn (J.S.); wangf121@163.com (F.W.); fangqi@zju.edu.cn (Q.F.); chu@zju.edu.cn (G.Y.)

**Keywords:** *Brachymeria lasus*, *Galleria mellonella*, parasitoid venom, cellular immunity, humoral immunity, cell death

## Abstract

The pupal endoparasitoid *B. lasus* injects venom into its host *G. mellonella* during oviposition, yet knowledge about the venom remains limited. This study explores how parasitism and venom from *B. lasus* impair the host’s cellular and humoral immunity. At 12–24 h post-parasitization, parasitized *G. mellonella* pupae had significantly lower total hemocyte counts and also exhibited higher mortality than non-parasitized controls. The proportion of plasmatocytes decreased, while the percentage of granulocytes increased. Parasitism also suppressed *in vitro* hemocyte spreading, with no significant difference in melanization between parasitized and control groups. Venom treatment significantly inhibited hemocyte spreading and increased cell mortality. Notably, venom-exposed hemocytes showed elevated reactive oxygen species levels and calcium ion concentrations, along with a significant decrease in mitochondrial membrane potential, while caspase 3 activity remained unchanged. These results suggest that both *B. lasus* parasitism and its venom suppress the cellular immunity of *G. mellonella* and have strong hemocytotoxic effects. The findings emphasize the role of venom in disrupting host defenses for the development of parasitoid offspring.

## Simple Summary

The parasitic wasp *Brachymeria lasus* (Hymenoptera: Chalcididae) has a broad host range, encompassing over 100 species across Lepidoptera, Hymenoptera, and Diptera. During oviposition, it injects venom into its host to enhance the survival and development of its offspring within the host body. However, the interactions between *B. lasus* and its hosts, as well as the biochemical composition and physiological functions of its venom, have received little attention. Our research focused on how *B. lasus* modulates host immunity, using *Galleria mellonella* as a model host. We discovered that both parasitism by *B. lasus* and its venom alone suppress cellular immunity in *G. mellonella* and exhibit strong hemocyte toxicity. This finding provides a foundation for in-depth analysis of *B. lasus* venom function and its role in regulating host immunity, holding potential for downstream development as a biopesticide.

## 1. Introduction

Parasitoid wasps represent a critical component in the natural regulation of insect pest populations, offering immense ecological and economic value, particularly in the context of sustainable biological control strategies [1,2]. These insects are remarkably diverse, with the global diversity of parasitoid wasps conservatively estimated to range from 500,000 to over one million species [3]. The success of parasitoid wasps in overcoming host defenses and ensuring the development of their offspring is primarily mediated through an arsenal of specialized parasitism-associated factors. These include venom [4,5,6,7], polydnaviruses (PDVs) [8,9,10,11], virus-like particles [12], ovarian proteins [13,14], and teratocytes [15,16,17], among others. In parasitoid species that lack PDVs, venom assumes a particularly central role in host manipulation. Parasitoid venom acts through a multitude of complex mechanisms, including the following: (1) disruption of host physiological homeostasis and metabolic regulation, (2) alteration of host development and growth trajectories [18,19,20], (3) suppression of immune responses [21,22], and (4) induction of paralysis [23,24]. For instance, the venom component Ae-γ-glutamyl transpeptidase (Ae-γ-GT) from *Aphidius ervi* induces host castration and causes host ovary degeneration [18]. In the ectoparasitoid *Scleroderma guani* (Hymenoptera: Bethylidae), a serine proteinase homolog (SguaSPH) inhibits phenoloxidase activity in the hemolymph of *Ostrinia furnacalis* [21]. Additionally, a serpin splicing isoform (PpS1V) in *Pteromalus puparum* venom suppresses host prophenoloxidase (PPO) activation [22]. Further examples include the *Tetrastichus brontispae* venom protein Tb4CL4-like, which inhibits cell spreading and encapsulation; *Microplitis mediator* MmGAP1, a cytoskeleton-disrupting factor; and *Pimpla hypochondriaca* venom proteins VPrl and VPr3, which suppress cellular aggregation [25,26,27,28]. These effects synergistically create a highly conducive internal environment for the survival and maturation of the parasitoid larvae.

Recent advancements in high-throughput proteomic and transcriptomic technologies have significantly accelerated the identification and functional characterization of venom components, thereby deepening our understanding of host–parasitoid interactions, particularly those involving immune suppression mediated by venom proteins [19,20,29]. The insect immune system, which serves as the primary barrier against parasitoid invasion, comprises two major arms: the humoral and cellular immune responses [30]. Humoral immunity involves the secretion of various soluble immune effectors into the hemolymph, including synthesis of antimicrobial peptides, hemolymph coagulation, and the melanization activated by phenoloxidase (PO) cascade [31]. Due to the effectiveness of the PO pathway in mounting a rapid and localized response against parasitoid eggs, venom from numerous parasitoids has evolved to specifically suppress this pathway. This is exemplified by the venoms of *Scleroderma guani* [21], *P*. *puparum* [22], *Cotesia rubecula* [32], and *Microplitis mediator* [33], among others.

In parallel with humoral immune evasion, parasitoid venoms exert profound effects on the cellular branch of host immunity. These effects encompass the inhibition of hemocyte spreading behavior, suppression of encapsulation responses, modulation of hemocyte populations, interference with phagocytic activity, and, in some cases, direct cytotoxicity against immune cells [2,5]. Considerable research has focused on how venom impacts hemocyte-mediated immune defenses, particularly the encapsulation and spreading capabilities of hemocytes [34,35,36,37,38,39,40,41,42,43,44,45]. The total number, diversity, and mortality rates of host hemocytes vary considerably depending on the species of parasitic wasp, reflecting the diverse immune evasion strategies employed by these insects. The venom of *Nasonia vitripennis* induces pronounced cytotoxic effects, directly leading to hemocyte death and a marked reduction in their overall numbers in the host hemolymph [39]. However, parasitism by *Asobara tabida* does not alter the hemocyte count in its host, as its eggs circumvent encapsulation not through venom activity but by virtue of the adhesive, fibrous nature of their outer eggshell, which prevents immune recognition and subsequent attack [46,47,48,49]. *Cotesia chilonis* venom has been observed to initially increase hemocyte counts in its host, though this effect does not persist into later stages of parasitism and does not significantly impact overall hemocyte viability or mortality rates [50]. Moreover, venoms from several other parasitoid species, including *Leptopilina* spp., *Meteorus pulchricornis*, *N. vitripennis*, *P. puparum*, and *Pimpla turionellae*, have all been demonstrated to induce hemocyte death, underscoring a shared cytotoxic function among phylogenetically diverse taxa [36,37,38,42,45,51].

*B. lasus* (Hymenoptera: Chalcididae), a generalist endoparasitoid, parasitizes more than 100 species spanning the insect orders Lepidoptera, Hymenoptera, and Diptera [52]. Despite its broad ecological relevance and apparent host plasticity, research on *B. lasus* has primarily concentrated on ecological interactions and host range studies [53,54,55], with little attention paid to the biochemical composition or physiological function of its venom. Given its generalist nature and taxonomic position within the underexplored Chalcididae family, *B. lasus* presents an ideal candidate for venom-based studies aimed at identifying novel immune modulators and developing next-generation biological control agents. Concurrently, *G*. *mellonella* poses a significant threat to apiculture, infesting honeybee hives [56,57] and causing economic losses estimated in the millions annually across China, Europe, and other regions [58,59]. Notably, *G. mellonella* serves as a well-established model organism [60,61], with its innate immune system and molecular mechanisms being thoroughly characterized [62]. 

Building on this foundation, the current study aimed to elucidate the immunomodulatory mechanisms exerted by *B. lasus* parasitism and venom in *G. mellonella*. We demonstrated that *B. lasus* venom induces significant hemocyte death in the host, thereby compromising cellular immunity and creating a favorable environment for larval development. To further investigate the molecular basis of this cytotoxicity, we quantitatively assessed hallmark indicators of programmed cell death (PCD). These parameters provided robust evidence for venom-induced apoptosis-like cell death, revealing mechanistic insights into how *B. lasus* manipulates host immunity to its advantage. Collectively, our findings expand the current understanding of host–parasitoid immune interactions and highlight *B. lasus* venom as a promising subject for future mechanistic studies. This work lays the groundwork for deeper functional dissection of venom components and their roles in immune suppression, with potential downstream applications in biopesticide development. Furthermore, our results contribute to a broader theoretical framework for understanding the co-evolutionary arms race between parasitoids and their hosts, particularly within the underexplored but ecologically important Chalcididae family.

## 2. Materials and Methods

### 2.1. Insect Collection, Rearing, and Parasitization

The greater wax moth, *G. mellonella*, was sourced from the Shanghai Ruiqing Fishing Bait Store and maintained under standardized laboratory conditions. Only pupae were selected for experimental use. The parasitoid *B. lasus* was propagated as a stable laboratory colony by continuously rearing field-collected individuals, using *G. mellonella* pupae as the exclusive host substrate. Both insect species were maintained at 25 °C, with 60% relative humidity and a photoperiod of 14:10 h (light/dark).

For parasitism assays, newly formed *G. mellonella* pupae (<24 h post-pupation) were exposed to mated female *B. lasus* individuals aged 5 days. Parasitism was confirmed through direct observation of ovipositor insertion into the host cuticle. After a standardized exposure period of 30 min, parasitoids were removed to ensure uniform parasitism conditions. Parasitized pupae were subsequently maintained under the same environmental parameters as described above.

### 2.2. Venom Collection

Venom extraction was conducted following a previously established protocol [50] with slight modifications to optimize yield and purity. Briefly, female *B. lasus* wasps were surface-sterilized using 75% ethanol, then rinsed thoroughly with sterile 0.01 M phosphate-buffered saline (1 × PBS, pH 7.4). The ovipositor complex, including venom glands and reservoirs, was carefully dissected from the terminal abdominal segment. Venom reservoirs were transferred into individual PBS droplets under a stereomicroscope. The venom-containing PBS solution was then collected into 1.5 mL microcentrifuge tubes and centrifuged at 12,000× *g* for 10 min at 4 °C. The resulting supernatant, enriched in venom components, was aliquoted and stored at −80 °C for downstream analyses. 1 VRE/µL represents the content of one venom reservoir equivalent collected in 1 μL of 1 × PBS (pH 7.4).

### 2.3. Hemocyte Quantification, Mortality Assessment, and Cell Spreading Assay

Total hemocyte count (THC), differential hemocyte count (DHC), hemocyte mortality, and cell spreading ability were evaluated at multiple time points. For the assessment of parasitization effects: Experimental groups were divided into parasitized and non-parasitized treatments. All parasitism-response assays were performed using *G. mellonella* hemolymph collected at 2, 4, 8, 12, and 24 h post-parasitism to enable comparative temporal analysis of immune modulation. For the analysis of venom effects: Test groups received venom at final concentrations of 0.06, 0.03, 0.01, and 0.005 VRE/µL, with 1 × PBS (pH 7.4) and bovine serum albumin (BSA) serving as negative controls. These methods were modified from [50].

Hemocyte collection: *G. mellonella* pupae were first surface-sterilized with 75% ethanol. Hemolymph was collected by piercing the wing bud cuticle with a sterile insect pin and diluted 10-fold in anticoagulant buffer (composition: 98 mM NaOH, 186 mM NaCl, 17 mM Na_2_EDTA, 41 mM citric acid, pH 4.5), modified from [63].

THC was determined using a hemocytometer under an inverted fluorescence microscope. Cells within five fixed grid squares (center and four corner squares) of the central counting area were quantified per replicate.

For DHC and cell spreading analyses, a mix of TC-100 insect culture medium containing 10% (*v*/*v*) fetal bovine serum (FBS), 1 μL phenylthiourea (PTU, 2.0 mmol/L), and 1 μL penicillin–streptomycin solution (10,000 U/mL penicillin; 10,000 μg/mL streptomycin) per 100 μL mix was prepared. Then, 20 μL of 10 × hemolymph was mixed with 100 μL of this mix and incubated in 96-well plates. DHC and cell spreading analyses were conducted in 96-well plates. Cells were visualized and photographed under an inverted fluorescence microscope, sampling five non-overlapping fields per well across three replicate wells. Proportions of granulocytes and plasmatocytes, as well as their spreading behavior, were quantified after 1 h. For venom-treated samples, observations were made at 0.5, 1, 2, and 4 h post-treatment. Hemocyte morphology was classified following [64]: in the non-spreading state, granulocytes (~20 µm in diameter) retained cytoplasmic granules with high refractivity, while plasmatocytes appeared round (~10 µm) and lacked granules. During spreading: granulocytes displayed peripheral actin cytoskeleton redistribution, and plasmatocytes adopted fibroblast-like, elongated morphologies.

Hemocyte mortality was determined using a CellTox™ Green Cytotoxicity Assay Kit (Promega, Madison, WI, USA). Fresh hemolymph with hemocytes was collected and diluted 10-fold in ice-cold 1 × PBS (pH 7.4). Aliquots (80 µL) of TC-100 medium supplemented with 10% FBS, 1 µL of penicillin–streptomycin (10,000 U/mL penicillin; 10,000 μg/mL streptomycin), 1 µL of PTU (2.0 mmol/L) and a CellTox™ Green dye (1000 × final dilution) were dispensed into 96-well plates. Following this, 10 µL of 10 × diluted hemolymph was added to each well and incubated in the dark at 27 °C for 1 h before imaging using the SS200 imaging system. The ratio of dead to total cells was then quantified.

### 2.4. Reactive Oxygen Species (ROS) Quantification

Intracellular ROS levels were quantified using a ROS Assay Kit (Beyotime Biotechnology, Shanghai, China). Test groups received venom at final concentrations of 0.01 VRE/µL. Hemocytes within hemolymph were extracted by gently piercing the insect cuticle with a sterile needle, allowing passive flow to minimize contamination, and immediately transferred to a PSB-coated tube. Hemocytes were incubated with DCFH-DA working solution (10 μM, diluted 1000-fold from 10 mM stock) at 27 °C under light-protected conditions for 8 h. As a positive control, a subset of hemocytes was pre-treated with Rosup (50 μg/mL, diluted 1000-fold from 50 mg/mL stock), a known ROS inducer, for 30 min prior to staining. Fluorescence intensity was measured using excitation and emission wavelengths of 488 nm and 525 nm, respectively, to quantify ROS accumulation.

### 2.5. Intracellular Calcium Ion Detection

Cytosolic calcium ion concentrations were measured using a calcium-sensitive fluorescent dye Fluo-4 AM (Beyotime Biotechnology, Shanghai, China). Test groups received venom at final concentrations of 0.01 VRE/µL. Hemocytes within hemolymph were extracted by gently piercing the insect cuticle with a sterile needle. Hemocytes were stained with Fluo-4 AM (1:1000 dilution) and incubated in the dark at 27 °C for 8 h. Following gentle removal of excess dye, fluorescence intensity was immediately recorded at Ex_488_/Em_525_ using a microplate reader to capture real-time Ca^2+^ flux.

### 2.6. Caspase-3 Activity and Mitochondrial Membrane Potential (MMP) Assay

Caspase-3 activity and mitochondrial membrane potential were assessed using a Mito-Tracker Deep Red 633 dye provided in the Live Cell Caspase-3 Activity and MMP Assay Kit (Beyotime Biotechnology, Shanghai, China). Test groups received venom at final concentrations of 0.01 VRE/µL. After an 8 h incubation with venom or control solutions, the supernatant was removed, hemocytes were washed, then treated with a Mito-Tracker working solution and a GreenNuc™ Caspase-3 Substrate (1:200 dilution). Following a 50 min incubation at room temperature, MMP fluorescence was detected at excitation/emission wavelengths of 622 nm/648 nm, respectively, to evaluate MMP depolarization and Caspase-3 activity fluorescence was detected at excitation/emission wavelengths of 500 nm/530 nm.

### 2.7. Hemocyte Encapsulation Assay

To evaluate the effects of parasitism and venom cellular encapsulation, we adopted the protocol from [65], with minor modifications. Briefly, 190 μL of TC-100 medium supplemented with 10% FBS, 1 μL of saturated phenylthiourea (PTU) solution and 1 μL penicillin–streptomycin solution (10,000 U/mL penicillin; 10,000 μg/mL streptomycin) was added to each well of a 48-well plate. Subsequently, 10 μL of PBS containing approximately 20 Sephadex A-25 beads and 10 μL of fresh hemolymph were added per well. For parasitism-treated samples, the plate was incubated at 27 °C for 1 h, and the extent of encapsulation was observed microscopically. For venom-treated samples, encapsulation assays were conducted at 0.5, 1, 2, and 4 h post-treatment using the same protocol. Test groups received venom at final concentrations of 0.06, 0.03, 0.01, and 0.005 VRE/µL, with phosphate-buffered saline (PBS) and bovine serum albumin (BSA) serving as negative controls. The encapsulation percentage was calculated based on the extent to which Sephadex A-25 beads were encapsulated.

### 2.8. Melanization Assay

Melanization activity triggered by parasitism was assessed following the method described by [50], with further refinements to improve sensitivity and reproducibility. Hemolymph were extracted by gently piercing the insect cuticle with a sterile needle. Briefly, hemolymph extracted from *G. mellonella* pupae was first diluted 10-fold with phosphate-buffered saline (PBS) and centrifuged at 3000× *g* for 10 min at 4 °C. The resulting cell-free supernatant was carefully collected for subsequent enzymatic analysis. 5 μL of *Micrococcus luteus* (0.1 μg/μL) was combined with 95 μL of the diluted hemolymph and gently agitated at 700 rpm for 5 min to ensure uniform mixing. Subsequently, 100 μL of freshly prepared L-DOPA (3 mg/mL), the chromogenic substrate for PO, was rapidly added to each sample. Absorbance at 490 nm was recorded at 5 min intervals over a 2 h period using a microplate reader. The rate of melanin formation, indicative of PO activity, was calculated based on the linear portion of the absorbance-time curve. Protein concentrations in hemolymph samples were determined using the Bradford assay, and PO activity was expressed in photometric units, where 1 unit corresponded to a change of 0.001 in OD_490_ per minute per milligram of hemolymph protein.

A complementary melanization assay was also conducted using a modified protocol based on the method of [22], which allowed for a more detailed evaluation of venom-induced immune suppression. Hemolymph was diluted 4-fold with Tris-buffered saline (TBS) and centrifuged under identical conditions (3000× *g*, 10 min, 4 °C) to remove cellular debris and obtain a clear supernatant. The reaction mixtures were assembled in a 384-well plate. To determine the influence of wasp venom on PPO activation, 5 μL aliquots of either BSA, PBS, PTU (2.0 mmol/L) or venom solutions (final concentrations of 0.05, 0.1, or 0.2 VRE/μL) were first mixed with 10 μL of diluted hemolymph in 384-well plates. Subsequently, 5 μL of *M. luteus* (0.1 μg/μL) and 5 μL of L-DOPA were added to each well, resulting in a final reaction volume of 25 μL. The PPO (prophenoloxidase) cascade was subsequently activated. After thorough mixing, absorbance at 470 nm (PO activity) was monitored every 5 min for a duration of 2 h at 25 °C. All measurements were performed in triplicate to ensure consistency and statistical robustness.

### 2.9. Data Analysis

All experimental data were subjected to rigorous statistical analysis to determine significance and ensure reproducibility. For comparisons between two treatment groups, Student’s *t*-test was applied. For analyses involving three or more groups, one-way analysis of variance (ANOVA) followed by Tukey’s post hoc multiple comparison test was employed to identify statistically significant differences. All analyses were performed using GraphPad Prism version 9.5 (GraphPad Software, San Diego, CA, USA). Data were presented as mean ± standard error of the mean (SE), and a *p*-value of <0.05 was considered statistically significant unless otherwise specified.

## 3. Results

### 3.1. Effects of Parasitism on THCs, DHCs, and Mortality

**Figure 1:**
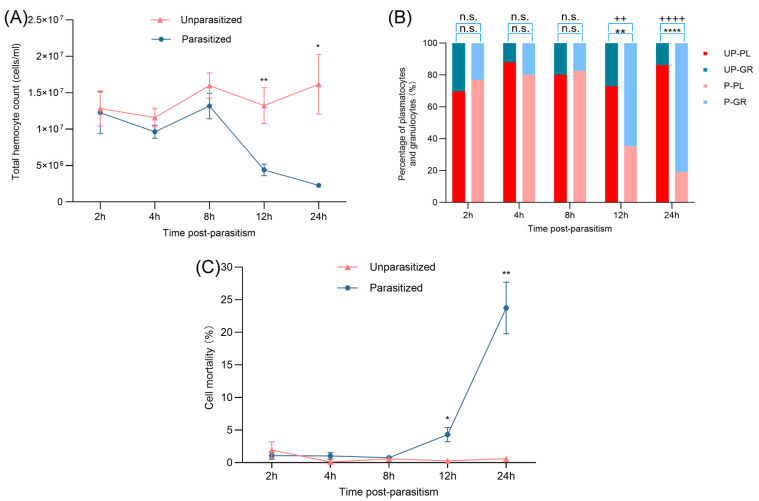
Effects of parasitism on THCs, DHCs, and mortality of *G. mellonella* pupae. (**A**) Total hemocyte counts (THCs) in *G*. *mellonella* pupae following parasitism. Values represent the mean ± SE (*n* = 5). Statistically significant differences between parasitized and unparasitized groups at the same time point were determined using Student’s *t*-test (* *p* < 0.05, ** *p* < 0.01). (**B**) Proportional changes in PL (plasmatocytes) and GR (granulocytes) over time post-parasitism. UP: unparasitized, P: parasitized. Data are expressed as mean ± SE (*n* = 3). Significant differences were assessed via Student’s *t*-test. “n.s.” indicates no statistically significant difference (*p* > 0.05), “++” indicates a statistically significant difference between UP-GR and P-GR (*p* ≤ 0.01), “++++” indicates a statistically significant difference between UP-GR and P-GR (*p* ≤ 0.0001), “**” indicates a statistically significant difference between UP-PL and P-PL (*p* ≤ 0.01), “****” indicates a statistically significant difference between UP-PL and P-PL (*p* ≤ 0.0001). (**C**) Hemocyte mortality in parasitized *G. mellonella* pupae. Data represent mean ± SE (*n* = 3). Significant differences were assessed via Student’s *t*-test (* *p* < 0.05, ** *p* < 0.01).

The dynamics of hemocyte populations in *G. mellonella* pupae following parasitism by *B. lasus* were first evaluated by examining total and differential hemocyte counts over time. From 2 to 8 h post-parasitism, the total hemocyte counts (THCs) of parasitized pupae remained comparable to those of their unparasitized counterparts (Figure 1A). However, a significant reduction in THCs was observed in the parasitized group beginning at 12 h, persisting through 24 h post-parasitism (Figure 1A). This temporal shift suggests a delayed but pronounced systemic immune modulation triggered by parasitism.

Analysis of differential hemocyte counts (DHCs) further highlighted parasitism-induced alterations in hemocyte composition. In unparasitized pupae, plasmatocytes consistently outnumbered granular hemocytes, maintaining a stable ratio throughout the 24 h observation period. In contrast, parasitized pupae exhibited a marked decline in plasmatocyte proportions, accompanied by a concurrent increase in granular hemocyte percentage beginning at 12 h and continuing through 24 h post-parasitism (Figure 1B). These changes reflect a parasitism-induced disruption in hemocyte subtype equilibrium, potentially as part of a host–parasite interaction strategy to suppress immune functions.

Moreover, parasitism significantly impacted hemocyte viability. A sharp increase in hemocyte mortality was observed in parasitized pupae, particularly from 12 to 24 h, compared to the unparasitized controls (Figure 1C). This finding implies that *B. lasus* parasitism not only alters hemocyte distribution but also compromises cell survival, thereby weakening the host immune response.

### 3.2. Effects of Parasitism on Hemocyte Spreading, Encapsulation, and Melanin Production

**Figure 2:**
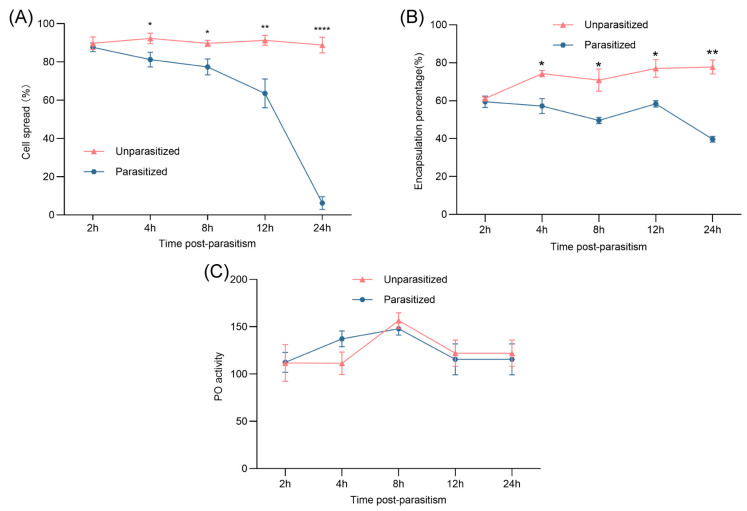
Effects of parasitism on hemocyte spreading, encapsulation response, and PO activity of *G. mellonella* pupae. (**A**) Impact of parasitism and related factors on hemocyte spreading in *G. mellonella* pupae. Data are shown as mean ± SE (*n* = 5). Statistical significance was determined by Student’s *t*-test (* *p* < 0.05, ** *p* < 0.01, **** *p* < 0.0001). (**B**) Effect of parasitism on the encapsulation response in *G. mellonella* pupae. Values are presented as mean ± SE (*n* = 3). Differences were analyzed using Student’s *t*-test (* *p* < 0.05, ** *p* < 0.01). (**C**) PO activity in hemolymph post-parasitism. Data are expressed as mean ± SE (*n* = 5). Student’s *t*-test was used to evaluate significance.

Hemocyte spreading, an essential aspect of cellular immunity, was significantly impaired following parasitism. In unparasitized pupae, over 88% of plasmatocytes and granulocytes adhered to substrate surfaces, forming filopodia or lamellipodia and exhibiting robust spreading behavior throughout the 24 h. In contrast, parasitized hemocytes exhibited clear spreading deficiencies as early as 2 h post-parasitism. By 24 h, only approximately 6% of hemocytes from parasitized pupae retained spreading capability (Figure 2A), suggesting an early and sustained suppression of cellular activation by *B. lasus*.

From 4 to 24 h post-parasitism, the encapsulation percentage of parasitized pupae remained substantially lower, ranging between 40 and 60%, compared to the consistently higher values (approximately 70–80%) in unparasitized controls (Figure 2B).Interestingly, PO activity did not significantly differ between parasitized and unparasitized groups across the experimental time points. (Figure 2C).

### 3.3. Effects of Venom on Hemocyte Spreading Behavior

**Figure 3:**
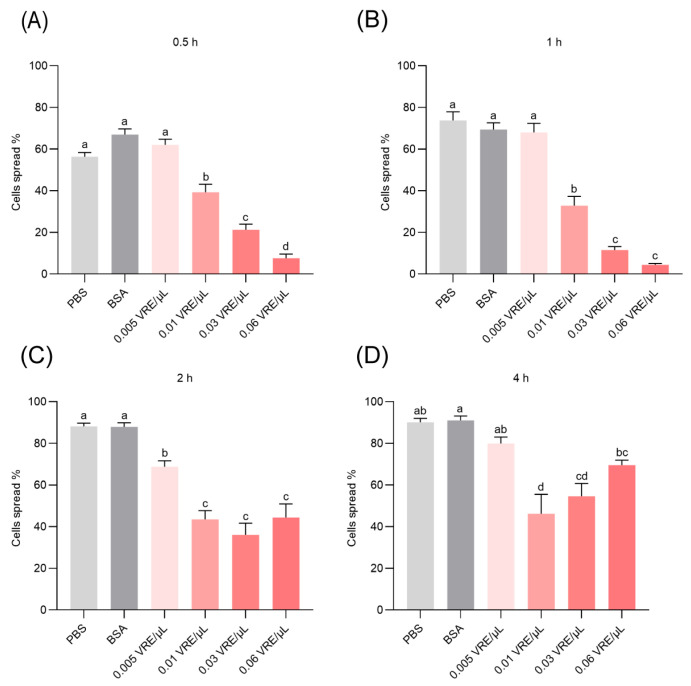
Effect of *B*. *lasus* venom on hemocyte spreading in *G. mellonella* pupae. Spreading ability was measured at 0.5, 1, 2 and 4 h (**A**–**D**). Data are expressed as mean ± SE (*n* = 6). Significant differences among treatment groups at each time point were determined using one-way ANOVA followed by Tukey’s post hoc test (*p* < 0.05). Distinct letters indicate statistically significant differences.

Exposure of hemocytes to *B. lasus* venom *in vitro* induced a rapid and sustained suppression of cell spreading activity. Significant inhibition was evident at all evaluated time points between 0.5 and 4 h post-treatment, with venom-treated groups exhibiting markedly reduced spreading compared to both PBS and BSA control groups (Figure 3A–D). Notably, this inhibitory effect was dose-dependent and most pronounced during the early phases of venom exposure (0.5–1 h; Figure 3A,B). These findings suggest that venom components directly interfere with cytoskeletal rearrangements or signaling pathways essential for hemocyte adhesion and spreading.

### 3.4. Effects of Venom on Encapsulation Response

**Figure 4:**
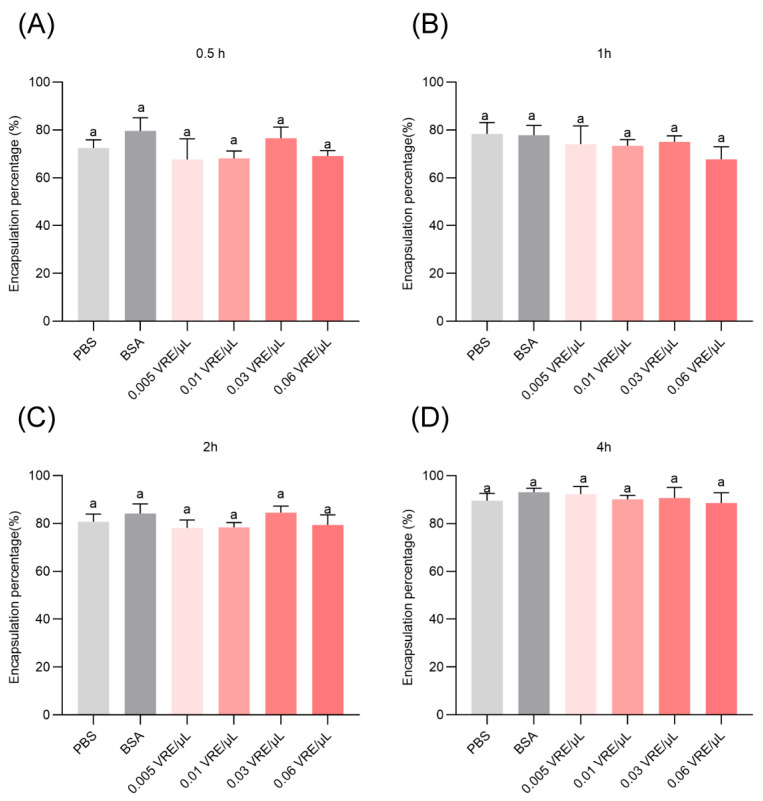
Effect of *B. lasus* venom on the encapsulation ability of hemocytes in *G. mellonella* pupae. Encapsulation ability was measured at 0.5, 1, 2, and 4 h (**A**–**D**). Values represent mean ± SE (*n* = 3). One-way ANOVA with Tukey’s multiple comparison test was used to determine statistical significance at *p* < 0.05. The same letters indicate no statistically significant differences (*p* > 0.05).

Notably, direct venom exposure *in vitro* did not produce statistically significant encapsulation suppression under the tested conditions. Across a range of concentrations (0.005–0.06 VRE/μL) and observation periods (0.5–4 h), no statistically significant differences in encapsulation were observed when compared to PBS or BSA controls (Figure 4A–D).

### 3.5. Effects of Venom on Melanin Formation

**Figure 5:**
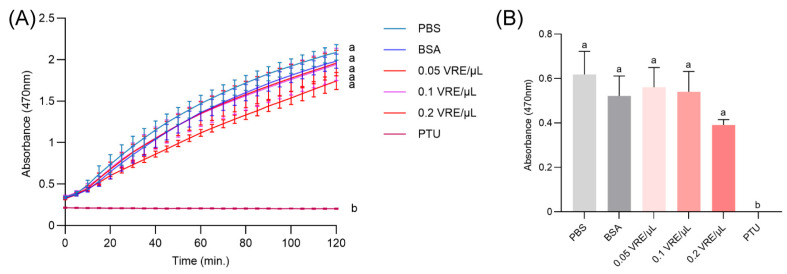
Effect of *B. lasus* venom on PO activity of *G. mellonella* hemolymph from pupae. (**A**) None of the tested venom doses (0.05, 0.1, or 0.2 VRE/μL) suppressed host hemolymph PO activity. Dopa chrome or dopamine chrome (melanization intermediates) was monitored at A_470_ every 5 min for 2 h. (**B**) Summary of absorbance A_470_ at 30 min. Data are presented as mean ± SE (*n* = 3). One-way ANOVA followed by Tukey’s post hoc test was applied to identify significant differences (*p* < 0.05). Distinct letters indicate statistically significant differences.

No significant difference in hemolymph melanization was observed across all tested venom doses (0.05, 0.1, or 0.2 VRE/μL) compared to PBS or BSA controls (Figure 5A). To standardize the comparison, absorbance values at the 30 min time point were selected for statistical analysis, indicating no significant difference in melanization between venom-exposed and control groups *in vitro* (Figure 5B). These results align with the *in vivo* findings in Section 3.2 and collectively suggest that venom from *B. lasus* does not directly suppress enzymatic components of the melanization cascade.

### 3.6. Effects of Venom on Hemocyte Death and Cell Death Mechanisms

To further understand the cytotoxic potential of *B. lasus* venom, hemocyte viability and cell death pathways were systematically assessed. Beginning at 4 h post-treatment, hemocyte mortality showed a marked increase in venom-exposed groups compared to controls, highlighting the pro-death activity of venom constituents (Table 1).

**Figure 6:**
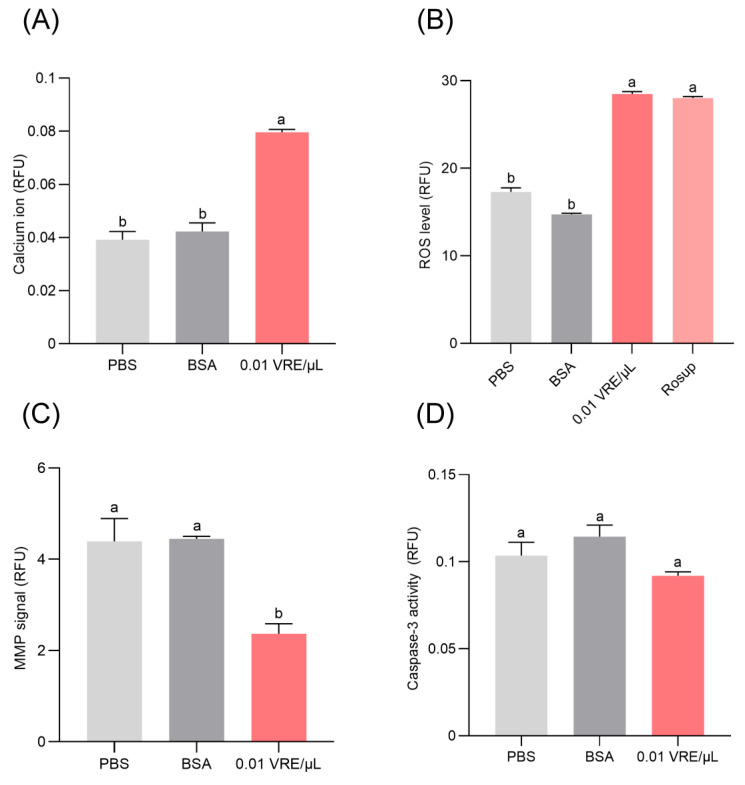
Various cell death-associated signaling pathways after venom treatment. (**A**) Intracellular calcium ion levels in *G. mellonella* hemocytes after venom treatment. (**B**) Reactive oxygen species (ROS) production. Rosup was used as a ROS-positive control. (**C**) Mitochondrial membrane potential (MMP). (**D**) Caspase-3 activity in hemocytes. All data are expressed as mean ± SE (*n* = 3). One-way ANOVA followed by Tukey’s post hoc test was applied to identify significant differences (*p* < 0.05). Distinct letters indicate statistically significant differences.

To delineate the mechanistic basis of venom-induced cell death, key cellular stress and apoptotic markers were evaluated, including mitochondrial membrane potential (MMP), intracellular calcium ion concentration, reactive oxygen species (ROS) accumulation, and caspase-3 activity. After 8 h of exposure to 0.01 VRE/µL venom, hemocytes exhibited significant MMP decline, elevated calcium levels, and increased ROS production relative to PBS and BSA controls (Figure 6A–C). However, no significant change was observed in caspase-3 activity (Figure 6D), suggesting that venom-induced hemocyte death may proceed through caspase-independent mechanisms such as necrosis or alternative programmed cell death pathways. These findings offer new insights into the cytotoxic action of *B. lasus* venom and its role in immune suppression during parasitism.

## 4. Discussion

The innate immune system of insects plays a pivotal role in defending against parasitoid wasps, engaging both cellular and humoral branches of immunity [30]. To achieve successful parasitism, parasitoid wasps must subvert or suppress the host’s immune defenses [2,5], particularly by modulating hemocyte populations—both in terms of absolute numbers and relative composition—and by disrupting key hemocyte-mediated functions such as adhesion, spreading, and encapsulation [35,66,67,68]. Since the density and functional capacity of hemocytes are fundamental determinants of immune competency in insects [30], it is unsurprising that parasitism often entails pronounced alterations in THCs, DHCs, and hemocyte morphology and behavior [69].

Our study revealed that parasitism by the endoparasitoid wasp *B. lasus* caused a significant decline in the THCs of *G. mellonella* pupae within 12 h post-infestation, with the reduction in plasmatocyte populations being especially pronounced. A prior investigation involving *P. puparum* also demonstrated a significant reduction in the proportion of host plasmatocytes post-parasitism, coupled with a compensatory rise in granular hemocytes [38]. The THC of *G. mellonella* pupae decrease was accompanied by a notable increase in hemocyte mortality *in vivo*. *In vitro* exposure of host hemocytes to crude venom extracted from *B. lasus* corroborated these findings, with venom-treated cells exhibiting a sharp rise in mortality. These observations collectively suggest that *B. lasus* venom acts as a potent parasitic factor that suppresses the host’s cellular immune function, at least in part, through the induction of hemocyte death. Similar venom-induced cytotoxic effects have been reported in various other parasitoid wasps [36,37,38,42,45,51].

In addition to altering hemocyte populations, both parasitization and venom treatment from *B. lasus* were found to significantly inhibit hemocyte spreading behavior. This aligns with prior studies that have reported venom-mediated suppression of hemocyte spreading [34,39]. Our results demonstrate that the encapsulation percentage of hemocytes in the parasitism group was lower than in the non-parasitism group. We propose that this primarily stems from parasitism-induced drastic hemocyte depletion, which reduces cell numbers sufficiently to create measurable differences from unparasitized controls. Conversely, no significant difference in encapsulation was observed with venom treatment alone despite its inhibition of hemocyte spreading. We speculate this apparent discrepancy may arise because the high hemocyte concentrations used *in vitro* assays potentially compensate for functional impairments. Therefore, *B. lasus* venom likely achieves encapsulation suppression predominantly through reducing hemocyte availability rather than direct functional inhibition. The precise mechanisms merit further investigation. The mechanistic basis of this retained functionality warrant further study. Intriguingly, neither parasitism nor venom exposure significantly affected PO activity or the melanization response of the host hemolymph. Our data also demonstrate a significant reduction in host hemocyte counts during *B. lasus* parasitism. Since most phenoloxidases are synthesized by hemocytes and their destruction activates the phenoloxidase cascade, it is possible that the parasitoid’s venom partially blocks the PO cascade, explaining why we do not observe a sharp increase in PO activity. This would be beneficial to the parasitoid, as it would allow it to resist secondary infections. The mechanistic underpinnings of how *B. lasus* modulates host humoral immunity remain an open question and merit further exploration.

Programmed cell death (PCD), as a significant type of cell death, has been extensively investigated [70,71]. Apoptosis has been identified as the primary mechanism through which various parasitoid wasp venoms induce host cell death. To better characterize the cell death pathway induced by *B. lasus* venom, we analyzed multiple cellular biomarkers, including intracellular calcium levels, mitochondrial membrane potential (MMP), reactive oxygen species (ROS) accumulation, and caspase-3 activity. Our results indicate that venom treatment for 8 h significantly elevated ROS production and intracellular calcium concentrations while concurrently diminishing mitochondrial membrane potential. These effects are hallmarks of oxidative stress-mediated PCD. Determining the specific modality of this programmed cell death, such as necroptosis or parthanatos, will require future detailed molecular analyses.

## 5. Conclusions

In summary, this study underscores the complex cellular immune interactions between the endoparasitoid *B. lasus* and its host, *G. mellonella*. Our findings demonstrate that *B. lasus* venom profoundly alters host immune cell populations and behaviors, including significant reductions in plasmatocyte and granulocyte abundance and spreading ability. These immune disruptions appear to be mediated through a programmed cell death process characterized by mitochondrial dysfunction, ROS accumulation, and calcium dysregulation. While the precise nature and molecular regulators of this venom-induced cell death remain to be elucidated, our study contributes important insights into parasitoid–host immune dynamics and highlights the potential of *B. lasus* venom as a biocontrol agent. Further mechanistic investigations into the venom’s molecular targets and pathways will not only enhance our understanding of parasitoid immunomodulation but also inform the development of novel, biologically based pest management strategies.

## Figures and Tables

**Table 1:** The mortality (%) of hemocytes affected by venom dosage.

Treatment	Dose *	Time (Hour) **						
		0.5	1	2	4	8	12	24
PBS	-	0.16 ± 0.16 a	0.45 ± 0.25 a	0.41 ± 0.22 c	0.63 ± 0.21 c	1.88 ± 0.70 c	1.65 ± 0.77 c	5.65 ± 0.89 c
BSA	-	1.38 ± 0.56 a	1.17 ± 0.40 a	1.27 ± 0.39 c	1.15 ± 0.43 c	1.64 ± 0.65 c	2.65 ± 0.77 c	6.18 ± 1.12 c
Venom	0.005	0.40 ± 0.40 a	0.53 ± 0.31 a	1.32 ± 1.15 c	1.82 ± 0.72 c	41.28 ± 9.82 b	86.96 ± 1.89 a	96.57 ± 0.08 a
	0.01	0.52 ± 0.52 a	1.26 ± 0.67 a	1.56 ± 0.84 bc	4.54 ± 0.51 bc	57.60 ± 2.90 ab	84.36 ± 1.85 ab	92.34 ± 1.47 ab
	0.03	0.0 ± 0.0 a	1.36 ± 0.2 a	2.41 ± 1.24 ab	11.33 ± 1.1 ab	63.26 ± 2.91 a	81.65 ± 0.82 ab	91.84 ± 0.41 ab
	0.06	0.81 ± 0.46 a	2.03 ± 1.24 a	2.67 ± 1.81 a	11.81 ± 3.19 a	64.08 ± 3.45 a	78.99 ± 1.34 b	89.17 ± 0.64 b

* Dose is in wasp equivalents (see Materials and Methods). ** Data (%) are expressed as mean ± SE (*n* = 3). Different lowercase letters indicate significant differences between different treatments within the same time point (*p* < 0.05).

## Data Availability

The data that support the findings of this study are available from the corresponding author upon reasonable request.

## References

[1] Condon M.A., Scheffer S.J., Lewis M.L., Wharton R., Adams D.C., Forbes A.A. (2014). Lethal interactions between parasites and prey increase niche diversity in a tropical community. Science.

[2] Asgari S., Rivers D.B. (2011). Venom proteins from endoparasitoid wasps and their role in host-parasite interactions. Annu. Rev. Entomol..

[3] Burke G.R., Sharanowski B.J. (2024). Parasitoid wasps. Curr. Biol..

[4] Mrinalini, Werren J.H., Malhotra A. (2017). Parasitoid wasps and their venoms. Evolution of Venomous Animals and Their Toxins.

[5] Quicke D.L.J., Butcher B.A. (2021). Review of venoms of non-polydnavirus carrying ichneumonoid wasps. Biology.

[6] Kosman E.S., Yaroslavtseva O.N., Kryukova N.A., Rotskaya U.N., Glupov V.V., Kryukov V.Y. (2025). Wax moth larvae demonstrate a high level of humoral immunity after envenomation by parasitoid *Habrobracon hebetor*. Entomol. Exp. Appl..

[7] Kamiyama T., Shimada-Niwa Y., Mori H., Tani N., Takemata-Kawabata H., Fujii M., Takasu A., Katayama M., Kuwabara T., Seike K. (2025). Parasitoid wasp venoms degrade *Drosophila* imaginal discs for successful parasitism. Sci. Adv..

[8] Beckage N.E. (2012). Polydnaviruses as endocrine regulators. Parasitoid viruses.

[9] Wang Z.Z., Ye X.Q., Shi M., Li F., Wang Z.H., Zhou Y.N., Gu Q.J., Wu X.T., Yin C.L., Guo D.H. (2018). Parasitic insect-derived miRNAs modulate host development. Nat. Commun..

[10] Wang Z.H., Zhou Y.N., Yang J., Ye X.Q., Shi M., Huang J.H., Chen X.X. (2021). Genome-wide profiling of *Diadegma semiclausum* Ichnovirus integration in parasitized *Plutella xylostella* hemocytes identifies host integration motifs and insertion sites. Front. Microbiol..

[11] Strand M.R. (2012). Polydnavirus gene products that interact with the host immune system. Parasitoid Viruses.

[12] Suzuki M., Tanaka T. (2006). Virus-like particles in venom of *Meteorus pulchricornis* induce host hemocyte apoptosis. J. Insect Physiol..

[13] Salvia R., Scieuzo C., Grimaldi A., Fanti P., Moretta A., Franco A., Varricchio P., Vinson S.B., Falabella P. (2021). Role of ovarian proteins secreted by *Toxoneuron nigriceps* (Viereck) (Hymenoptera, Braconidae) in the early suppression of host immune response. Insects.

[14] Salvia R., Cozzolino F., Scieuzo C., Grimaldi A., Franco A., Vinson S.B., Monti M., Falabella P. (2022). Identification and functional characterization of *Toxoneuron nigriceps* ovarian proteins involved in the early suppression of host immune response. Insects.

[15] Strand M.R. (2014). Teratocytes and their functions in parasitoids. Curr. Opin. Insect Sci..

[16] Pinto C.P.G., Walker A.A., Robinson S.D., King G.F., Rossi G.D. (2022). Proteotranscriptomics reveals the secretory dynamics of teratocytes, regulators of parasitization by an endoparasitoid wasp. J. Insect Physiol..

[17] Wu Z.W., Yuan R.Z., Gu Q.J., Wu X.T., Gu L.C., Ye X.Q., Zhou Y.A., Huang J.H., Wang Z.Z., Chen X.X. (2023). Parasitoid serpins evolve novel functions to manipulate host homeostasis. Mol. Biol. Evol..

[18] Russo E., Di Lelio I., Shi M., Becchimanzi A., Pennacchio F. (2023). *Aphidius ervi* venom regulates Buchnera contribution to host nutritional suitability. J. Insect Physiol..

[19] Scieuzo C., Salvia R., Franco A., Pezzi M., Cozzolino F., Chicca M., Scapoli C., Vogel H., Monti M., Ferracini C. (2021). An integrated transcriptomic and proteomic approach to identify the main *Torymus sinensis* venom components. Sci. Rep..

[20] Nakamatsu Y., Tanaka T. (2003). Venom of ectoparasitoid, *Euplectrus* sp. near *plathypenae* (Hymenoptera: Eulophidae) regulates the physiological state of *Pseudaletia separata* (Lepidoptera: Noctuidae) host as a food resource. J. Insect Physiol..

[21] Wu C.Y., Huang J.M., Zhao Y.J., Xu Z.W., Zhu J.Y. (2020). Venom serine proteinase homolog of the ectoparasitoid *Scleroderma guani* impairs host phenoloxidase cascade. Toxicon.

[22] Yan Z.C., Fang Q., Liu Y., Xiao S., Yang L., Wang F., An C.J., Werren J.H., Ye G.Y. (2017). A venom serpin splicing isoform of the endoparasitoid wasp *Pteromalus puparum* suppresses host prophenoloxidase cascade by forming complexes with host hemolymph proteinases. J. Biol. Chem..

[23] Yu K.L., Chen J., Bai X., Xiong S.J., Ye X.H., Yang Y., Yao H.W., Wang F., Fang Q., Song Q.S. (2023). Multi-omic identification of venom proteins collected from artificial hosts of a parasitoid wasp. Toxins.

[24] Cantori L.V., Garcia A.G., Pinto A.D., Godoy W.A.C., Parra J.R.P. (2022). Detailed look at paralysis of hosts by the ectoparasitoid *Habrobracon hebetor* (Hymenoptera: Braconidae): Does more efficient paralysis mean more effective parasitism?. BioControl.

[25] Zhang X.M., Zhang H.J., Liu M., Liu B., Zhang X.F., Ma C.J., Fu T.T., Hou Y.M., Tang B.Z. (2019). Cloning and immunosuppressive properties of an acyl-activating enzyme from the venom apparatus of *Tetrastichus brontispae* (Hymenoptera: Eulophidae). Toxins.

[26] Du J., Lin Z., Volovych O., Lu Z.Q., Zou Z. (2020). A RhoGAP venom protein from *Microplitis mediator* suppresses the cellular response of its host *Helicoverpa armigera*. Dev. Comp. Immunol..

[27] Dani M.P., Richards E.H. (2009). Cloning and expression of the gene for an insect haemocyte anti-aggregation protein (VPr3) from the venom of the endoparasitic wasp, *Pimpla hypochondriaca*. Arch. Insect Biochem. Physiol..

[28] Dani M.P., Richards E.H. (2010). Identification, cloning, and expression of a second gene (vprI) from the venom of the endoparasitic wasp, *Pimpla hypochondriaca* that displays immunosuppressive activity. J. Insect Physiol..

[29] Burke G.R., Strand M.R. (2014). Systematic analysis of a wasp parasitism arsenal. Mol. Ecol..

[30] Strand M.R. (2008). The insect cellular immune response. Insect Sci..

[31] Zdybicka-Barabas A., Stączek S., Kunat-Budzyńska M., Cytryńska M. (2025). Innate immunity in insects: The lights and shadows of phenoloxidase system activation. Int. J. Mol. Sci..

[32] Asgari S., Zhang G.M., Zareie R., Schmidt O. (2003). A serine proteinase homolog venom protein from an endoparasitoid wasp inhibits melanization of the host hemolymph. Insect Biochem. Mol. Biol..

[33] Zhou L.Z., Wang R.J., Lin Z., Shi S.K., Chen C.H., Jiang H.B., Zou Z., Lu Z.Q. (2023). Two venom serpins from the parasitoid wasp Microplitis mediator inhibit the host prophenoloxidase activation and antimicrobial peptide synthesis. Insect Biochem. Mol. Biol..

[34] Richards E.H., Parkinson N.M. (2000). Venom from the endoparasitic wasp *Pimpla hypochondriaca* adversely affects the morphology, viability, and immune function of hemocytes from larvae of the tomato moth, *Lacanobia oleracea*. J. Invert. Pathol..

[35] Furihata S.X., Matsumoto H., Kimura M.T., Hayakawa Y. (2013). Venom components of *Asobara japonica* impair cellular immune responses of host *Drosophila melanogaster*. Arch. Insect Biochem. Physiol..

[36] Wang J.L., Yan Z.C., Xiao S., Wang B.B., Fang Q., Schlenke T., Ye G.Y. (2021). Characterization of a cell death-inducing endonuclease-like venom protein from the parasitoid wasp *Pteromalus puparum* (Hymenoptera: Pteromalidae). Pest Manag. Sci..

[37] Zhang Z., Ye G.Y., Cai J., Hu C. (2005). Comparative venom toxicity between *Pteromalus puparum* and *Nasonia vitripennis* (Hymenoptera: Pteromalidae) toward the hemocytes of their natural hosts, non-target insects and cultured insect cells. Toxicon.

[38] Cai J., Ye G.Y., Hu C. (2004). Parasitism of *Pieris rapae* (Lepidoptera: Pieridae) by a pupal endoparasitoid, *Pteromalus puparum* (Hymenoptera: Pteromalidae): Effects of parasitization and venom on host hemocytes. J. Insect Physiol..

[39] Rivers D.B., Ruggiero L., Hayes M. (2002). The ectoparasitic wasp *Nasonia vitripennis* (Walker) (Hymenoptera: Pteromalidae) differentially affects cells mediating the immune response of its flesh fly host, *Sarcophaga bullata* Parker (Diptera: Sarcophagidae). J. Insect Physiol..

[40] Uçkan F., Er A., Ergin E. (2010). Levels of encapsulation and melanization in *Galleria mellonella* (Lepidoptera: Pyralidae) parasitized and envenomated by *Pimpla turionellae* (Hymenoptera: Ichneumonidae). J. Appl. Entomol..

[41] Mortimer N.T., Goecks J., Kacsoh B.Z., Mobley J.A., Bowersock G.J., Taylor J., Schlenke T.A. (2013). Parasitoid wasp venom SERCA regulates *Drosophila* calcium levels and inhibits cellular immunity. Proc. Natl. Acad. Sci. USA.

[42] Kim-Jo C., Gatti J.-L., Poirié M. (2019). *Drosophila* cellular immunity against parasitoid wasps: A complex and time-dependent process. Front. Physiol..

[43] Yang L., Wang B.B., Qiu L.M., Wan B., Yang Y., Liu M.M., Wang F., Fang Q., Stanley D.W., Ye G.Y. (2019). Functional characterization of a venom protein calreticulin in the ectoparasitoid *Pachycrepoideus vindemiae*. Insects.

[44] Kryukova N.A., Dubovskiy I.M., Chertkova E.A., Vorontsova Y.L., Slepneva I.A., Glupov V.V. (2011). The Effect of *Habrobracon hebetor* venom on the activity of the prophenoloxidase system, the generation of reactive oxygen species and encapsulation in the haemolymph of *Galleria mellonella* larvae. J. Insect Physiol..

[45] Zhang G.M., Schmidt O., Asgari S. (2006). A calreticulin-like protein from endoparasitoid venom fluid is involved in host hemocyte inactivation. Dev. Comp. Immunol..

[46] Eslin P., Prévost G. (2000). Racing against Host’s Immunity Defenses: A likely strategy for passive evasion of encapsulation in *Asobara tabida* parasitoids. J. Insect Physiol..

[47] Kraaijeveld A.R., Alphen J.M. (1994). Geographical variation in resistance of the parasitoid *Asobara tabids* against encapsulation by *Drosophila melanogaster* larvae: The mechanism explored. Physiol. Entomol..

[48] Kraaijeveld A.R., Nowee B., Najem R.W. (1995). Adaptive variation in host-selection behaviour of *Asobara tabida*, a parasitoid of *Drosophila* larvae. Funct. Ecol..

[49] Eslin P., Giordanengo P., Fourdrain Y., Prévost G. (1996). Avoidance of encapsulation in the absence of VLP by a braconid parasitoid of *Drosophila* larvae: An ultrastructural study. Can. J. Zool..

[50] Teng Z.W., Xu G., Gan S.Y., Chen X., Fang Q., Ye G.Y. (2016). Effects of the endoparasitoid *Cotesia chilonis* (Hymenoptera: Braconidae) parasitism, venom, and calyx fluid on cellular and humoral immunity of its host *Chilo suppressalis* (Lepidoptera: Crambidae) larvae. J. Insect Physiol..

[51] Er A., Uçkan F., Rivers D.B., Ergin E., Sak O. (2010). Effects of parasitization and envenomation by the endoparasitic wasp *Pimpla turionellae* (Hymenoptera: Ichneumonidae) on hemocyte numbers, morphology, and viability of its host *Galleria mellonella* (Lepidoptera: Pyralidae). Ann. Entomol. Soc. Am..

[52] Tian S., Gu T.Z., Chen C., Zhao X.D., Liu P.C., Hao D.J. (2021). The effects of temperature and host size on the development of *Brachymeria lasus* parasitising *Hyphantria cunea*. J. For. Res..

[53] Husni, Kainoh Y., Honda H. (2001). Effects of host pupal age on host preference and host suitability in *Brachymeria lasus* (Walker) (Hymenoptera: Chalcididae). Appl. Entomol. Zool..

[54] Mao H.X., Kunimi Y. (1994). Effects of temperature on the development and parasitism of *Brachymeria lasus*, a pupal parasitoid of *Homona magnanima*. Entomol. Exp. Appl..

[55] Mao H.X., Kunimi Y. (1994). Longevity and fecundity of *Brachymeria lasus* (Walker) (Hymenoptera: Chalcididae), a pupal parasitoid of the oriental tea tortrix, *Homona magnanima* Diakonoff (Lepidoptera: Tortricidae) under laboratory conditions. Appl. Entomol. Zool..

[56] Kumar Y., Yadav S. (2018). Seasonal incidence of greater wax moth, *Galleria mellonella* Linnaeus in *Apis mellifera* colonies in ecological condition of Hisar. J. Entomol. Zool. Stud..

[57] Sohail M., Aqueel M.A., Ellis J.D., Raza A.M., Ullah S. (2020). Consumption, digestion, and utilization of beeswax by greater wax moths (*Galleria mellonella* L.). J. Apicult. Res..

[58] García N.L. (2018). The current situation on the international honey market. Bee World.

[59] Hosni E.M., Al-Khalaf A.A., Nasser M.G., Abou-Shaara H.F., Radwan M.H. (2022). Modeling the potential global distribution of honeybee pest, *Galleria mellonella* under changing climate. Insects.

[60] Banfi D., Bianchi T., Mastore M., Brivio M.F. (2024). Optimization of experimental infection of the animal model *Galleria mellonella* Linnaeus 1758 (Lepidoptera: Pyralidae) with the Gram-positive bacterium *Micrococcus luteus*. Insects.

[61] Cutuli M.A., Petronio G.P., Vergalito F., Magnifico I., Pietrangelo L., Venditti N., Di Marco R. (2019). *Galleria mellonella* as a consolidated in vivo model hosts: New developments in antibacterial strategies and novel drug testing. Virulence.

[62] Wojda I. (2017). Immunity of the greater wax moth *Galleria mellonella*. Insect Sci..

[63] Stoepler T.M., Castillo J.C., Lill J.T., Eleftherianos I. (2012). A Simple Protocol for Extracting Hemocytes from Wild Caterpillars. J. Vis. Exp. JoVE.

[64] Mizerska-Dudka M., Andrejko M. (2014). *Galleria mellonella* hemocytes destruction after infection with *Pseudomonas aeruginosa*. J. Basic Microbiol..

[65] Ling E.J., Yu X.Q. (2006). Cellular encapsulation and melanization are enhanced by immulectins, pattern recognition receptors from the tobacco hornworm *Manduca sexta*. Dev. Comp. Immunol..

[66] Mabiala-Moundoungou A.D.N., Doury G., Eslin P., Cherqui A., Prévost G. (2010). Deadly venom of *Asobara japonica* parasitoid needs ovarian antidote to regulate host physiology. J. Insect Physiol..

[67] Labrosse C., Eslin P., Doury G., Drezen J.M., Poirié M. (2005). Haemocyte changes in *Drosophila melanogaster* in response to long gland components of the parasitoid wasp *Leptopilina boulardi*: A Rho-GAP protein as an important factor. J. Insect Physiol..

[68] Labrosse C., Stasiak K., Lesobre J., Grangeia A., Huguet E., Drezen J.M., Poirie M. (2005). A RhoGAP protein as a main immune suppressive factor in the *Leptopilina boulardi* (Hymenoptera, Figitidae)—*Drosophila melanogaster* interaction. Insect Biochem. Mol. Biol..

[69] Stettler P., Trenczek T., Wyler T., Pfister-Wilhelm R., Lanzrein B. (1998). Overview of parasitism associated effects on host haemocytes in larval parasitoids and comparison with effects of the egg-larval parasitoid *Chelonus inanitus* on its host *Spodoptera littoralis*. J. Insect Physiol..

[70] Yuan J.Y., Ofengeim D. (2024). A guide to cell death pathways. Nat. Rev. Mol. Cell Biol..

[71] Qian S.E., Long Y., Tan G.L., Li X.G., Xiang B., Tao Y.G., Xie Z.Z., Zhang X.W. (2024). Programmed cell death: Molecular mechanisms, biological functions, diseases, and therapeutic targets. MedComm.

